# Real-Life Benefit of OCT Imaging for Optimizing PCI Indications, Strategy, and Results

**DOI:** 10.3390/jcm8040437

**Published:** 2019-03-30

**Authors:** Dan Mircea Olinic, Mihail Spinu, Calin Homorodean, Mihai Claudiu Ober, Maria Olinic

**Affiliations:** 1 Medical Clinic No. 1, “Iuliu Hatieganu” University of Medicine and Pharmacy, Cluj-Napoca 400006, Romania; dolinic@yahoo.com (D.M.O.); maria_olinic@yahoo.com (M.O.); 2 Interventional Cardiology Department, Emergency Clinical Hospital, Cluj-Napoca 400006, Romania; mihai_ober@yahoo.com

**Keywords:** optical coherence tomography, percutaneous coronary interventions, borderline lesions, nonsignificant lesions, left main, bifurcations

## Abstract

Background: The aim of this study was to evaluate the benefit of standard practice Optical Coherence Tomography (OCT) imaging, as a complement to coronary angiography (CA), for optimizing the indications, strategy, and results of percutaneous coronary interventions (PCI). Methods: We retrospectively analyzed 182 patients with OCT imaging in a single tertiary center. Results: OCT use had a low prevalence (3.1% of 4256 CAs and 1.7% of 3027 PCIs). OCT was used post-CA in 71.5% and post-PCI in 28.5% of cases, mainly in acute coronary syndromes—95.6%. OCT was performed for borderline lesions in 43.4% of cases; lesion severity was reassessed as severe and led to PCI in 64.5% of them. OCT was performed for nonsignificant lesions in 17% of cases; lesion severity was reassessed as severe and led to PCI in 38.7% of them. OCT provided optimal selection for PCI strategy in 11% of cases. OCT identified suboptimal PCI results in 54% left main PCIs and in 48% bifurcation PCIs with optimal CA; PCI optimization was performed. In the only seven patients with suboptimal PCI, OCT revealed an optimal result in four cases, thus avoiding unneccessary optimization. In 27.3% of patients with post-CA OCT and PCI result “systematic” OCT control, a PCI optimization was indicated. Conclusion: OCT supplied a major benefit in 86.2% of cases, especially by identifying significant coroanry stenosis in CA borderline and nonsignificant lesions; OCT led to PCI indication in two-thirds and, respectively, one-third of these cases. In the post-PCI context, OCT led to an indication of PCI optimization in half of the complex left main and bifurcation lesions, as well as in a quarter of “systematic” post-PCI OCT controls.

## 1. Introduction

Cardiovascular diseases represent a major health burden, with coronary artery disease (CAD) as the most important cause of morbidity and mortality [1,2]. Every effort is made for improving CAD diagnosis, identifying patients with an indication for revascularization and optimal revascularization treatment through either interventional cardiology or cardiac surgery, resulting in a progressive decrease in CAD mortality [3,4].

Coronary angiography (CA) is the gold standard invasive procedure currently used for CAD diagnosis, decision-taking and assessing efficacy of percutaneous coronary interventions (PCIs) [5]. CA limitations are related to suboptimal ability to identify complicated atherosclerotic plaques and sometimes even to incorrect grading of the severity of coronary stenosis [5].

Optical coherence tomography (OCT) further improves invasive intracoronary imaging, offering new insights on the coronary wall and lumen, due to its high resolution (10 micrometers) [3,6]. OCT provides accurate characterization of atherosclerotic plaques, in terms of severity and extension, and identifies high-risk and complicated plaques, as well as coronary thrombi [5,6].

Over the last ten years, fast acquisition OCT devices and software allowed this method to be used safely, in everyday practice, as a complement to CA. OCT is therefore not only a very well recognized in vivo CAD research tool [6,7,8], but also an increasingly used real-life tool. Various indications have been proposed for OCT use in current clinical practice [9,10,11], as experience with OCT use has grown [12,13,14]. Recent guidelines already recommend OCT use for optimizing stent-related PCI procedures [15].

The aim of this study is to evaluate the benefit of standard practice OCT imaging, as a complement to CA for optimizing the indications, strategy and results of percutaneous coronary interventions. The study covers five years of practice of a single tertiary center from Romania.

## 2. Methods

### 2.1. Study Population

This study retrospectively analyzes the indications and benefit of OCT use in 182 patients, evaluated in a single tertiary center from Romania (Cluj County Emergency Hospital, Department of Interventional Cardiology), over a period of 5 years (January 2012–September 2017). During this interval, 4256 patients underwent CA for suspected CAD, of whom, 3027 patients also had PCI.

No exclusion criteria were considered. Informed consent was obtained before the invasive procedure. Clinical data and angiographic examinations were stored in the hospital database and in a dedicated catheterization laboratory database.

Data regarding cardiovascular risk factors [2] were obtained from patients history, physical examination, and medical records. Arterial hypertension was defined as known or newly diagnosed blood pressure values above 140/90 mmHg. Diabetes mellitus was defined as known or newly diagnosed blood fasting glucose above 126 mg/dL or glycated haemoglobin >6.5%. Dyslipidemia was defined as low-density lipoprotein cholesterol >130 mg/dL and/or triglycerides >150 mg/dL. Renal insufficiency was defined as a glomerular filtration rate <60 mL/min. Smoking habit was defined by current or recent smoking. Overweight was defined as body mass index >25 kg/m^2^.

CA was performed via right radial artery in 143 (78.6%) patients, via femoral artery in 35 (19.2%) patients and transbrachial in the remaining four (2.2%) patients, on Siemens Coroskop T.O.P and Siemens Artis Zee angiographs (Siemens Healthineers, Erlangen, Germany). Severity of coronary stenosis was assessed visually and by quantitative coronary analysis (QCA). The highest degree of stenosis severity, usually the area stenosis severity provided by QCA, was considered. After CA, coronary arteries were classified as normal (without plaques), with nonsignificant lesions (<50%), with borderline lesions (50–70%) or with significant stenosis (>70%).

### 2.2. OCT Acquisition Technique, Analysis, and Indications

OCT images were acquired with a commercially available system (ILUMIEN OPTIS OCT Intravascular Imaging System, St. Jude Medical, St. Paul, MN, USA), using an over the wire OCT catheter (C7 Dragonfly™ and Dragonfly™ OPTIS™, St. Jude Medical, St. Paul, MN, USA). The entire length of the region of interest was scanned using the integrated automated pullback device at 20 mm/s. During image acquisition, coronary blood flow was replaced by continuous flushing of contrast medium directly from the guiding catheter. All images were recorded digitally, stored, and analyzed using proprietary software (St. Jude Medical, St. Paul, MN, USA) in concordance with standard consensus of OCT use [5].

OCT was performed by all three senior interventional cardiologists, using the predefined indications in Table 1, during working hours and, two days each week, 24/24 h, for emergency situations like acute coronary syndromes (ACS).

Indications for OCT use were the ones generally accepted at the time of the investigation (Table 1) [5,10,16]. OCT was performed either after CA, for lesion evaluation or pre-PCI strategy assessment, or after PCI, for result assessment. OCT quantification of coronary lesion severity was made using the proprietary software, as area stenosis compared to largest pre-lesion reference area. An OCT area stenosis of at least 70% was considered as significant stenosis, with an indication for PCI.

The study complies with the Declaration of Helsinki on human research.

### 2.3. Statistical Analysis

Categorical variables were expressed as frequencies and compared with χ^2^ test. A two-sided *p*-value of 0.05 was considered significant.

## 3. Results

### 3.1. Demographic, Clinical and Angiographic Data

The investigated population includes 174 (95.6%) patients with ACS, of which 50 had ST-elevated myocardial infarction (STEMI), 39 had non-ST elevated myocardial infarction (NSTEMI), 85 had unstable angina (UA), and eight had stable CAD (SCAD). Demographic, clinical and angiographic data of OCT investigated patients are presented in Table 2 and data regarding their cardiovascular risk factors are presented in Table 3.

### 3.2. Post Coronarography OCT

**Figure 1:**
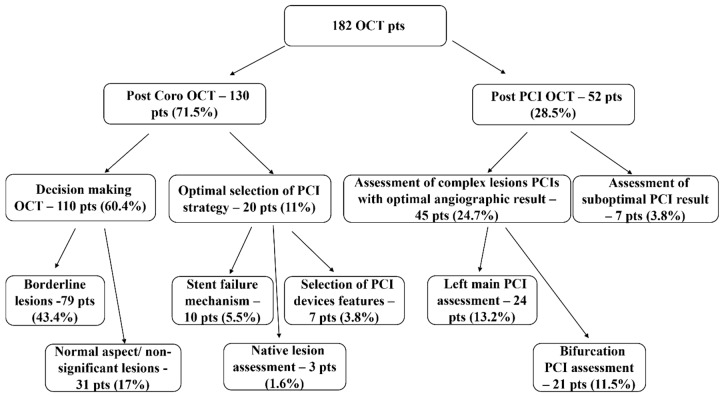
OCT indications. Pts: patients.

OCT was performed in two main instances: first, as a complemmentary imaging method after CA—130 patients (71.5% of the OCT investigations) and second, as an imaging method just used first after PCI—52 patients (28.5% of the OCT investigations) (Figure 1). Post-CA OCT was made in 3.1% of the total 4256 CAs performed in CAD patients, while post-PCI OCT was done in 1.7% of the total 3027 PCIs. OCT had two main indications, in the 130 patients post-CA group: OCT to provide accurate diagnosis and therapeutic decisions for revascularization (“decision-making OCT group”)—110 patients (60.4% of the OCT investigations)—and, respectively, OCT to provide optimal selection of PCI strategy—20 patients (11% of the OCT investigations) (Figure 1).

#### 3.2.1. “Decision-making OCT Group”

In the “decision-making OCT group” (110 patients), there were 79 patients (43.4% of the OCT investigations) with borderline (50–70%) CA lesions and 31 patients (17% of the OCT investigations) with nonsignificant lesions (<50%) or with a normal aspect on CA (Figure 1).

##### Borderline CA Lesions

**Figure 2:**
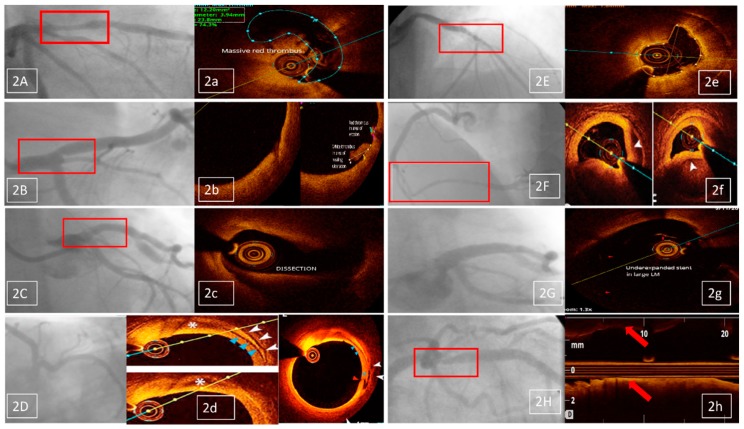
(**A**)—Anterior STEMI previously treated with fibrinolytic therapy—angiographically borderline lesion on proximal LAD, **a**—OCT shows massive red thrombus, with significant lumen stenosis; (**B**)—UA patient—angiographically nonsignificant lesion on ostial LAD, **b**—OCT shows signs of culprit lesion (white and red thrombus), with permeable lumen; (**C**)—Anterior STEMI patient—ostial LAD haziness of nonsignificant lesion, **c**—OCT shows coronary dissection, with significant lumen stenosis; (**D**)—Anterior STEMI patient—angiographically normal coronary aspect, **d**—OCT shows a dissection and hematoma originating from a vasa vasorum hemorrhage, being probably the underlying physiopathology of the ACS; (**E**)—NSTEMI patient at 10 years after a LCX bare metal stent implantation—angiographically intra-stent haziness, **e**—OCT shows neoatherosclerosis as the mechanism for stent failure; (**F**)—Inferior STEMI female patient—angiographically long, double lumen pattern, RCA stenosis, **f**—OCT confirms spontaneous dissection as the underlying physiopathology and guides PCI (guide wire presence in the true lumen and choice of stent’s “landing zones”); (**G**)—LM PCI with excellent angiographic result, **g**—OCT shows significant malapposition; (**H**)—Suboptimal angiographic result of proximal LAD stent placement, **h**—OCT shows excellent result.

In the 79 patients with a high clinical and electrocardiogram suspicion of CAD and with borderline CA lesions, in 77 of them with ACS (43 UAs, 17 STEMIs, and 17 NSTEMIs) and only two with SCADs an accurate decision for revascularisation could not be made based on CA alone, so OCT was performed. After performing OCT, the severity of coronary artery lesions was reassessed and graded as severe (>70%) (Figure 2A/a), with an indication for revascularization in 51 patients (64.5% of the 79 cases) (Table 4).

##### Nonsignificant Lesions or Normal Coronary Aspect

In the 31 patients with nonsignificant lesions (<50%) (Figure 2B/b and 2C/c) or with a normal coronary arteries aspect on CA (Figure 2D/d), there were 29 patients with ACS (15 STEMIs, 10 UAs, and four NSTEMIs). These ACS pts represented 15.3% of the total 189 ACS patients with nonsignificant lesions or normal coronary arteries investigated in our center, during the investigated time interval. There also were two SCAD patients. OCT identified 12 patients with significant (>70%) lesions (38.7% of the 31 cases), leading to PCI revascularization (Table 4).

When performed after CA, OCT led to revascularization in significantly more patients with borderline lesions than in ones with nonsignificant lesions or normal coronary arteries (64.5 vs. 38.7%, *p* = 0.013).

#### 3.2.2. OCT for Optimal Selections of PCI Strategy

The second main indication for OCT, after CA, was represented by patients in whom a decision for PCI revascularization was taken after CA, but OCT was still needed on top of CA, in order to provide optimal selection of PCI strategy (Figure 1). In these 20 patients (11% of the OCT investigations), there were three indications for OCT:

- identification of stent failure mechanism (10 patients, 5.5% of the OCT investigations); OCT proved restenosis in eight and neoatherosclerosis in two patients (Figure 2E/e);
- selection of PCI devices features (seven patients, 3.8% of the OCT investigations); there were three cases of long lesions PCI (Figure 2F/f), two of left main (LM) PCI and another two of bifurcation PCI;
- comprehensive assessment of native lesions suspected of complications (three patients, 1.6% of the OCT investigations); OCT confirmed complicated atherosclerotic plaques in all three of them, with identification of thrombus in one patient.

### 3.3. Post-PCI OCT Group

The “post-PCI” OCT group included 52 patients, with two main indications for imaging (Figure 1): assessment of complex lesions PCIs, with an optimal angiographic result—45 cases (24.7% of the OCT investigations) and assessment of suboptimal PCI results—seven cases (3.8%).

#### 3.3.1. Assessment of Complex Lesions PCIs with Optimal Angiographic Result Included:

##### Left Main Lesions PCI Assessment

This group included 24 patients with LM PCI, representing 13.2% of OCT indications and 19.8% of the 121 LM PCIs performed in our center, during the investigated time interval; there were mainly ACS (nine UA, eight STEMI, and six NSTEMI) patients, while two had SCAD; OCT was performed for LM PCI control in cases of technically challenging procedures (15 cases), two stents technique (seven cases), and for important LM, LAD, or LCX decalibration (two cases); optimal OCT results were found in 11 patients, without need for LM PCI optimization; OCT imaging found malappositions (10 patients) (Figure 2G/g) and edge dissections (three patients), leading to a decision for PCI optimization (with new balloon dilatation or, respectively, new stent implantation) in these 13 out of the 24 patients (54% of the LM PCIs with optimal angiographic results) (Table 4).

##### Bifurcation Lesions PCI Assessment

This group included 21 patients with bifurcation PCI, representing 11.5% of OCT indications and 2.2% of the 955 bifurcation PCIs performed in our center, during the investigated time interval; there were only ACS (11 UA, seven NSTEMI, and three STEMI) patients; OCT was performed for bifurcation PCI control in cases of technically challenging procedures (11 cases) and two stents techniques (10 cases); optimal OCT results were found in 11 patients, without need for PCI optimization; OCT imaging found malappositions (six patients) and edge dissections (four patients), leading to a decision for PCI optimization in these 10 out of the 21 patients (48% of the bifurcation PCIs with optimal angiographic results) (Table 4).

#### 3.3.2. Assessment of Suboptimal PCI Results

This group included seven patients (three STEMI, two NSTEMI, and two UA), representing only 0.23% of the 3027 PCIs performed in our center in the investigated time interval. The indication for OCT imaging was represented by

- angiographic suspicion of edge dissection (three cases); only one was confirmed by OCT, leading to PCI optimization;
- angiographic suspicion of extensive coronary dissection (one case)—unconfirmed by OCT;
- angiographic suspicion of coronary thrombus (two cases), confirmed by OCT in both cases and followed by PCI optimization;
- angiographic suspicion of significant residual stenosis in the native coronary, proximal to the stented lesion (one case), unconfirmed by OCT (Figure 2H/h).

In this group, OCT imaging revealed the presence of an optimal PCI result in four of the seven patients (57.1%), thus avoiding unneccessary PCI optimization. There were only three out of seven patients (42.8%) in whom PCI optimization was really needed.

### 3.4. Decision Change after OCT

For the entire group of 182 patients investigated by OCT, intracoronary imaging had a decisive benefit for optimizing interventional cardiology indications and strategy, in 157 patients (86.2%), as follows

- in all the 130 patients having OCT imaging as a complement to CA, for assessing the indication of revascularization and
- in 27 of the 52 patients investigated post-PCI (51.9% of this subgroup).

### 3.5. OCT Investigations not Included in This Study

There also were 44 OCT investigations performed after PCI, out of 77 cases, in patients from the “post-CA” group. In these patients, OCT catheter was already used before PCI, OCT investigations were therefore performed for PCI result control. In this group of patients, all post-PCI angiographic results were optimal, but OCT found suboptimal result and led to optimization in 12 cases (27.3%): 10 with balloon post-dilatation for stent underexpansion and two with new stent implantation for edge dissections.

## 4. Discussion

Multiple centers use OCT in a variety of clinical scenarios for diagnosis and therapeutic optimization in coronary interventions [9,10,11,12,13,14]. A recently issued European Association of Percutaneous Cardiovascular Interventions (EAPCI) expert consensus document on clinical use of intracoronary contained a broad array of OCT indications [10]. The ESC guidelines on myocardial revascularization [15] recommend OCT use for PCI optimization (Class IIa, Level of evidence B) and for identifying stent failure mechanism (Class IIa, Level of evidence C). A recent clinical practice survey [11] offered the perception of interventional cardiologists on the indications for OCT use and their relative prevalence. Our study provides actual evidence on real-life OCT use in a tertiary interventional cardiology center, allowing analysis of its indications and highlighting its benefits. The main benefit proved to be the identification of significant coronary stenosis, with an indication for PCI, in patients with borderline and nonsignificant coronary lesions on CA. Our study also proved OCT interest for optimizing imaging results after PCI, in complex LM and bifurcation lesions, as well as in a “systematic” post-PCI OCT control approach. This imagistic PCI optimization would translate into a better clinical outcome [5,10].

In our study, OCT proves particularly useful in the clinical setting of ACS (95.6% of the investigated patients), with 71.5% of OCTs performed as a complementary imaging method to CA, representing 3.1% of CAs in our center.

We mainly used OCT to provide accurate diagnosis and therapeutic decisions concerning revascularization (60.4% of the OCT investigations). A moderate proportion (43.4%) of the OCT investigations were performed for borderline lesions, and 17% for assessment of coronary arteries with nonsignificant lesions or with a normal aspect. Our approach is in consensus with the recent european recommendation [15] of OCT use in the up to 25% of NSTE-ACS patients with angiographically normal epicardial arteries, for identifying culprit lesions or rule out mechanisms such as dissection and haematomas (MI with nonobstructive coronary arteries, MINOCA). In ACS patients, physiological evaluation with fractional flow reserve has a limited value, with contradictory reports on safety [15,17,18] and unknown prognostic value [15].

A very important finding in our study is that OCT, performed complementary to CA, in ACS, reassessed the severity of coronary lesions and led to the indication of revascularization in 64.5% of borderline lesions, and in 38.7% of nonsignificant lesions or normal coronary arteries. This result would support inclusion of OCT imaging in the practice guidelines, in this setting.

In our study, OCT also proved useful for the control of LM PCI patients with optimal angiographic results, which accounted for 13.2% of OCT indications. In these patients, suboptimal OCT findings as malappositions and edge dissections led to PCI optimization in not less than 54% of the cases. This finding underlines the limits of angiography for LM PCI assessment and may support the need for a systematic OCT imaging in this setting. In our study, OCT was used in only 19.8% of LM PCI patients, with technically challenging procedures, a two-stent technique, or important LM, LAD, or LCX decalibration.This was in contrast to the survey on intravascular imaging use, where participants reported a 77% use for LM PCI guidance [11].

OCT was useful for optimization of bifurcation PCI, in difficult, technically challenging procedures and two-stent techniques. Despite a very good angiographic result in these patients, OCT found suboptimal results in 48% of the cases, with need for optimization. These findings support the limited existing data [19,20,21] regarding the need for more than angiography in bifurcation PCI. Other clinical practitioners reported a 53.5% use of IVI for guiding bifurcation PCI [11]. However, we used OCT for only 2.19% of our bifurcation PCIs (11.5% of total OCTs).

While other authors report that 79.6% of IVI evaluations are done for PCI strategy planing, in selected cases [11], this indication accounted for only 11% of OCTs, in our center. We used pre-PCI OCT for identifying stent failure mechanisms, selecting PCI devices features, and assesing native lesions.

OCT provided support in cases with suboptimal angiographic PCI results, in whom imaging proved in fact to correspond to a good PCI result, in four of the seven investigated patients. This is a rare OCT indication but its results further support that OCT imaging may overcome CA limitations.

While being available during the whole 5-year period of our study, OCT was used in only 1.7% of patients undergoing PCI. Our findings are comparable to the results of a recent large observational study, which showed a 1.3% prevalence of OCT use for PCI guiding [22].

Our relatively low prevalence of OCT use (3.1% of CA and 1.7% of PCIs) is similar to the approach of 16.6% of interventionalists in the recent clinical practice survey, that used intravascular imaging in less than 5% of their patients [11]. It is however to be noted that, in this same survey [11], 51.3% of the participants use IVI in more that 15% of patients, another 18% of participants use IVI in 5–15% of patients, and 10.3% of participants use IVI only in highly selected cases, while 3.9% of interventional cardiologists do not use any IVI [11].

Our study reports that OCT, while used in a limited number of pts submitted to CA and PCI, offer a substantial benefit for 86.2% of the investigated patients, leading to optimization of interventional cardiology indications and strategy. This is true in all patients in the post-CA group and in 51.9% of the post-PCI group.

Our results concerning OCT-related decision changes in the post-PCI LM and bifurcations complex lesions group (51.9%) are similar to the ones reported by the DOCTORS [23] trial, namely, a 50% change in procedural strategy in the OCT group. The ILUMIEN I study [24] reported 27% decision change in the post-PCI group. This low prevalence of OCT-related decision change post-PCI in the ILLUMIEN I study, can be explained by the systematic use of OCT, both pre- and post-PCI [24]. In our study, the systematic post-PCI use of OCT, in patients already having had OCT pre-PCI, led to identification of suboptimal PCI results, mainly underexpansion and edge dissection, with an indication for PCI optimization, in 27.3% of the cases, similar to the one in ILLUMIEN I study [24].

## 5. Study Limitations

Our study is an observational, retrospective, single-center one, with a limited sample size, without a statistical power calculation; thereby, our results should be interpreted with caution and considered hypothesis generating. Clinical follow-up of our patients is beyond the purpose of our study. Long term clinical benefit of a dual imagistic approach, using both CA and OCT, and not only CA, for the selection of PCI indications, strategy and result assessment and optimization, would require either a randomized study, or a comparison between centers using these two approaches.

## 6. Conclusions

The main OCT benefit is in borderline and nonsignificant lesions on CA, in whom OCT identifies significant coronary stenosis and leads to PCI indication in two-thirds and, respectively, one-third of the patients. In the post-PCI context, OCT leads to an indication of PCI optimization in half of the complex left main and bifurcation lesions, as well as in a quarter of “systematic” post-PCI OCT controls.

## Figures and Tables

**Table 1:** Indications for OCT use, adapted from Tearney G.J. et al. [5], Räber L. et al. [10] and Karanasos A. et al. [16]. OCT: optical coherence tomography; PCI: percutaneous coronary intervention; ACS- acute coronary syndrome.

OCT Indication	Specific Area of Interest
Post-Coronary Angiography and before a first PCI procedure	
Lesion evaluation	Culprit lesion evaluation in ACS patients without coronary angiography significant stenosis
	Evaluation of lesions with angiographic haziness (suspected dissection/thrombus/calcification)
Pre-PCI assessment	Measurements of lumen diameter and lesion length for PCI devices selection (balloon/stent dimensions)
	Lesion assessment for PCI technique/strategy selection (for left main and large bifurcations)
	Evaluation of “landing zones”
	Evaluation of guide wire position (in cases of coronary dissection/chronic occlusion)
Post-PCI	
Immediate assessment of PCI result	Evaluation of stent expansion (identification of under-expansion/residual stenosis)
	Evaluation of potential vascular injury (identification of edge dissection/intra-stent thrombus/tissue protrusion)
Late evaluation of suspected stent failure	Identification and characterization of restenosis
	Identification of in stent thrombosis
	Identification of neoatherosclerosis

**Table 2:** Demographic, clinical and angiographic data of patients investigated by OCT (LAD—left anterior descending artery, LCX—left circumflex artery, LM—left main, RCA—right coronary artery).

		STEMI (No. of Patients)	NSTEMI (No. of Patients)	UA (No. of Patients)	SCAD (No. of Patients)	Total (No. of Patients/%)
		50	39	85	8	182
**Gender**	**Male**	34	29	55	6	124 (68.1%)
	**Female**	16	10	30	2	58 (31.9%)
**Age**	**<40**	2	0	3	0	5 (2.7%)
	**≤40–49**	22	3	10	2	37 (20.3%)
	**≤50–59**	9	13	20	2	44 (24.2%)
	**≤60–69**	8	10	36	3	57 (31.3%)
	**≤70–79**	8	12	16	1	37 (20.3%)
	**≥80**	1	1	0	0	2 (1.1%)
**Coronary Angiography Results**	**Normal**	2	1	3	1	7 (3.8%)
	**LM**	5	6	11	0	22 (12.1%)
	**1 Vessel Disease**	29	8	30	2	69 (37.9%)
	**2 Vessels Disease**	6	7	17	3	33 (18.1%)
	**3 Vessels Disease**	8	17	24	2	51 (28%)
**OCT Examined Vessel**	**LM**	9	18	30	2	59 (32.4%)
	**LAD**	31	18	49	6	104 (57.1%)
	**LCX**	3	2	2	0	7 (3.8%)
	**RCA**	7	0	4	0	11 (6.1%)
	**Venous Graft**	0	1	0	0	1 (0.5%)

**Table 3:** Study population cardiovascular risk factors.

**Hypertension**	143 (78.6%)
**Diabetes mellitus**	46 (25.3%)
**Dyslipidemia**	105 (57.7%)
**Renal Insufficiency**	11 (6%)
**Smoking habit**	52 (28.6%)
**Overweight**	35 (19.2%)

**Table 4:** Treatment options after OCT.

Indications	Conservative Treatment No. of Patients/%	Revascularization No. of Patients/%	Total No. of Patients
Borderline lesions	28(35.5%)	51(64.5%)	79
Nonsignificant lesions or normal coronaries	19(61.3%)	12(38.7%)	31
Stent failure mechanism		10	10
Native lesion assessment		3	3
Selection of PCI devices		7	7
LM PCI control, after optimal angiography	11 (45.8%)	13 (54.2%)	24
Bifurcation PCI control, after optimal angiography	11 (52.3%)	10 (47.7%)	21
Suboptimal PCI result on angiography	4 (57.1%)	3 (42.9%)	7

## References

[1] (2018). Non-Communicable Diseases Country Profiles 2018. apps.who.int/iris/bitstream/handle/10665/274512/9789241514620-eng.pdf.

[2] Olinic D.M., Spinu M., Olinic M., Homorodean C., Tataru D.A., Liew A., Schernthaner G.H., Stanek A., Fowkes G., Catalano M. (2018). Epidemiology of peripheral artery disease in Europe: VAS Educational Paper. Int. Angiol..

[3] Kim Y., Johnson T.W., Akasaka T., Jeong M.H. (2018). The role of optical coherence tomography in the setting of acute myocardial infarction. J. Cardiol..

[4] Ha F.J., Giblett J.P., Nerlekar N., Cameron J.D., Meredith I.T., West N.E.J., Brown A.J. (2017). Optical Coherence Tomography Guided Percutaneous Coronary Intervention. Heart Lung Circ..

[5] Tearney G.J., Regar E., Akasaka T., Adriaenssens T., Barlis P., Bezerra H.G., Bouma B., Bruining N., Cho J.M., Chowdhary S. (2012). International Working Group for Intravascular Optical CoherenceTomography (IWG-IVOCT). Consensus standards for acquisition, measurement, and reporting of intravascular optical coherence tomography studies: A report from the International Working Group for Intravascular Optical Coherence Tomography Standardization and Validation. J. Am. Coll. Cardiol..

[6] Spînu M., Olinic D.M., Olinic M., Homorodean C. (2018). In vivo imaging of complicated atherosclerotic plaque-role of optical coherence tomography (OCT). Rom. J. Morphol. Embryol..

[7] Otsuka F., Joner M., Prati F., Virmani R., Narula J. (2014). Clinical classification of plaque morphology in coronary disease. Nat. Rev. Cardiol..

[8] Kini A.S., Vengrenyuk Y., Yoshimura T., Matsumura M., Pena J., Baber U., Moreno P., Mehran R., Maehara A., Sharma S. (2017). Fibrous cap thickness by optical coherence tomography in vivo. J. Am. Coll. Cardiol..

[9] Di Mario C., Mattesini A. (2018). Will Optical Coherence Tomography Become the Standard Imaging Tool for Percutaneous Coronary Intervention Guidance?. JACC Cardiovasc. Interv..

[10] Räber L., Mintz G.S., Koskinas K.C., Johnson T.W., Holm N.R., Onuma Y., Radu M.D., Joner M., Yu B., Jia H. (2018). Clinical use of intracoronary imaging. Part 1: Guidance and optimization of coronary interventions. An expert consensus document of the European Association of Percutaneous Cardiovascular Interventions. EuroIntervention.

[11] Koskinas K.C., Nakamura M., Räber L., Colleran R., Kadota K., Capodanno D., Wijns W., Akasaka T., Valgimigli M., Guagliumi G. (2018). Current use of intracoronary imaging in interventional practice—Results of a European Association of Percutaneous Cardiovascular Interventions (EAPCI) and Japanese Association of Cardiovascular Interventions and Therapeutics (CVIT) Clinical Practice Survey. EuroIntervention.

[12] Homorodean C., Ober M.C., Iancu A.C., Olinic M., Tataru D., Spinu M., Olinic D.M., Burzotta F., Trani C., Erglis A. (2017). How should I treat this mini-crush stenting complication?. EuroIntervention.

[13] Olinic D.M., Spinu M., Homorodean C., Olinic M. (2017). Vasa vasorum induced LAD dissection and haematoma in an anterior STEMI patient with nearly normal angiography: The role of OCT. Kardiol. Pol..

[14] Homorodean C., Spinu M., Ober M.C., Olinic M., Olinic D.M. (2017). Spontaneous coronary dissection: Optical coherence tomography insights before and after stenting. Cardiol. J..

[15] Neumann F.J., Sousa-Uva M., Ahlsson A., Alfonso F., Banning A.P., Benedetto U., Byrne R.A., Collet J.P., Falk V., Head S.J. (2019). 2018 ESC/EACTS Guidelines on myocardial revascularization. Eur. Heart J..

[16] Karanasos A., Ligthart J., Witberg K., van Soest G., Bruining N., Regar E. (2012). Optical Coherence Tomography: Potential Clinical Applications. Curr. Cardiovasc. Imaging Rep..

[17] Leone A.M., Cialdella P., Lassandro Pepe F., Basile E., Zimbardo G., Arioti M., Ciriello G., D’Amario D., Buffon A., Burzotta F. (2019). Fractional flow reserve in acute coronary syndromes and in stable ischemic heart disease: Clinical implications. Int. J. Cardiol..

[18] Hakeem A., Almomani A., Uretsky B.F. (2017). Role of fractional flow reserve in the evaluation and management of patients with acute coronary syndrome. Curr. Opin. Cardiol..

[19] Wolfrum M., De Maria G.L., Banning A.P. (2017). Optical coherence tomography to guide percutaneous treatment of coronary bifurcation disease. Expert Rev. Cardiovasc. Ther..

[20] Onuma Y., Okamura T., Muramatsu T., Uemura S., Serruys P.W. (2015). New implication of three-dimensional optical coherence tomography in optimising bifurcation PCI. EuroIntervention.

[21] Onuma Y., Katagiri Y., Burzotta F., Holm N.R., Amabile N., Okamura T., Mintz G.S., Darremont O., Lassen J.F., Lefèvre T. (2019). Joint consensus on the use of OCT in coronary bifurcation lesions by European and Japanese bifurcation clubs. EuroIntervention.

[22] Jones D.A., Rathod K.S., Koganti S., Hamshere S., Astroulakis Z., Lim P., Sirker A., O’Mahony C., Jain A.K., Knight C.J. (2018). Angiography Alone Versus Angiography Plus Optical Coherence Tomography to Guide Percutaneous Coronary Intervention: Outcomes From the Pan-London PCI Cohort. JACC Cardiovasc. Interv..

[23] Meneveau N., Souteyrand G., Motreff P., Caussin C., Amabile N., Ohlmann P., Morel O., Lefrançois Y., Descotes-Genon V., Silvain J. (2016). Optical coherence tomography to optimize results of percutaneous coronary intervention in patients with non-ST-elevation acute coronary syndrome: Results of the multicenter, randomized DOCTORS study (Does Optical Coherence Tomography Optimize Results of Stenting). Circulation.

[24] Wijns W., Shite J., Jones M.R., Lee S.W., Price M.J., Fabbiocchi F., Barbato E., Akasaka T., Bezerra H., Holmes D. (2015). Optical coherence tomography imaging during percutaneous coronary intervention impacts physician decision-making: ILUMIEN I study. Eur. Heart J..

